# Well-being issues: Its influence on RPE and enjoyment in masters water polo training and the player–coach gap

**DOI:** 10.1371/journal.pone.0340261

**Published:** 2026-01-13

**Authors:** Corrado Lupo, Damiano Li Volsi, Paolo Riccardo Brustio, Alexandru Nicolae Ungureanu

**Affiliations:** 1 NeuroMuscularFunction Research Group, School of Exercise and Sport Sciences, Department of Medical Sciences, University of Turin, Turin, Italy; 2 NeuroMuscularFunction Research Group, School of Exercise and Sport Sciences, Department of Clinical and Biological Sciences, University of Turin, Turin, Italy; 3 NeuroMuscularFunction Research Group, School of Exercise and Sport Sciences, Department of Life Sciences and Systems Biology, University of Turin, Turin, Italy; eCampus University: Universita degli Studi eCampus, ITALY

## Abstract

The present study aimed to monitor male master water polo players’ training by means of well-being (Hooper-Index), internal training load (ITL) parameters (rating of perceived exertion, RPE; session-RPE), and rate of enjoyment, for different types of training (i.e., swimming, SW; technical and tactical, TTW; training matches, TM; friendly matches; FM). Seventeen male master water polo players (age: 50 ± 12 years) performed 19 ± 7 sessions and reported Hooper-Index scores in the morning of training day, and RPE (CR-10) and rate of enjoyment after sessions. Linear mixed effects models were applied to quantify whether players’ RPE, session-RPE, and rate of enjoyment were: (i) different for type of training session, (ii) affected by pre-session well-being, and (iii) correlated to corresponding coach’s estimations. FM was the training type with the highest session-RPE (p < 0.001, ES range = 1.4–1.9), whereas SW reported the lowest rate of enjoyment (p < 0.05, ES range = 0.8–1.4). Similar effects emerged for coach’s estimations (the highest session-RPE in FM, p < 0.001, ES range = 1.4–2.1; the lowest rate of enjoyment in SW, p < 0.001, ES range = 3.2–5.3). RPE resulted affected by all well-being factors excepting sleep quality (β range = 0.16–0.28), whereas session-RPE was influenced only by fatigue, and Hooper-Index overall (β = 0.23) and no effect emerged for rate of enjoyment. Finally, players’ and coach’s ITL and rate of enjoyment were not correlated, with exception of TM session-RPE (β = 0.21). Master water polo coaches could benefit from these findings, being aware of how training load could be: different for types of workouts, influenced by pre-session well-being, and differently perceived by players if compared with their coach’s estimations.

## Introduction

The measurement of the internal training load (ITL) represents the most crucial reference to assess workout response [1]. In fact, this measure reflects the physiological response to the external training load and is able to make coaches aware of the effective training stimuli, thus having valuable information to program subsequent training sessions [2].

Several studies have successfully verified the reliability of the use of the rating of perceived exertion (RPE) and the session-RPE (i.e., the duration of training session multiplied by RPE) to quantify ITL. Although RPE is a subjective method, it has been satisfactory tested for being an ecological and reliable scale for measuring the exertion level in a training session, thanks to large correlations emerged with the gold standards such as Edwards’ HR-based methods [3–6] ITL has been studied in team sports, even considering official matches [7] or different types of training sessions [3,8], competition levels [4], and training purposes [9]. In addition, it was studied in relation to players’ perceptions and coaches’ estimations (of their player’s perceptions) to identify potential differences in how exertion is perceived [10]. Similarly, perceived well-being factors are able to influence acute neuromuscular performance or hormonal concentration [11], despite the relationship between session-RPE and well-being scores, as assessed by the four Hooper scales (i.e., sleep quality/disorders, stress, fatigue, and delayed onset muscle soreness [DOMS]), remains controversial [12]. Specifically, while ITL quantified using the session-RPE method resulted positively correlated with pre-session DOMS, the other three scales showed contrasting trends [13–15]. Differently, in female volleyball players, perceived ITL resulted influenced by pre-session well-being status [15]. Finally, sport enjoyment can be also considered as valuable parameter for monitoring training since it has been defined as “a positive affective response to the sport experience that reflects feelings and/or perceptions such as pleasure, liking, and experienced fun” [16].

Even though recent studies have been contributed to better understand swimming water polo patterns [17], scarce evidence-based information on water polo, about training monitoring or performance in general, is not so extended in literature. Session-RPE has been applied to monitor ITL of youth players (under-17 category) [3], reporting a general satisfactory grade of reliability considering correlations with Edwards HR-based method in different types of training sessions. More recently, RPE has been considered as a marker to enhance training and match monitoring as well as to assess recovery trends in a top-level national team preparing for an international championship [18]. Moreover, in a study on Italian first division water polo [7] session-RPE was applied to evaluate been the internal load perceived after single championship matches. In the same study, the Hooper Index has also been used to investigate players’ well-being thirty-six hours post-match, to verify if players’ optimal conditions were reestablished before the beginning of the first weekly training session; in addition, the overall enjoyment of the match activity has been recorded too. The findings of this study showed that ITL, well-being, and rate of enjoyment should not be used interchangeably, even though well-being resulted associable with RPE, and it tends to be also negatively influenced by perceived enjoyment values [7] More recently, despite for youth water polo players, ITL has been showed to be negatively correlated with fatigue, recovery capability, and well-being in general perceived after match, also highlighting how enjoyment is more perceived after official matches than friendly ones [19].

However, despite some studies considered well-being (Hooper Index) as both a prerequisite and a factor that potentially influences the next session’s internal training load (RPE) [15,20], and rate of enjoyment [20] no research has applied this approach to water polo training. In addition, the growing participation in master-level water polo (i.e., agonist level performed by over 30 years old players, according to specific age categories), which can be considered as positive counterbalance to any dropout phenomenon emerging along a sport career [21], has been demonstrated by the presence of national [22] and international championships [23]. Nevertheless, no studies have examined the training or competition performance for this age category. Finally, no study on water polo training has been considered to establish if players’ and coach’ ITL and enjoyment perceptions can be considered as coherently associable.

Therefore, for all the above mentioned lacks of literature, the present study on master water polo players aimed to establish whether: 1) RPE, session-RPE, and rate of enjoyment differ based on the type of training session (i.e., swimming sessions, SW; technical and tactical workouts, TTW; training matches, TM; and friendly matches, FM); 2) pre-session well-being level (i.e., Hooper Index) affects perceived ITL (i.e., RPE and session-RPE values) and enjoyment in relation to the type of training session; 3) players’ and coach’s perceptions (RPE, session-RPE, and rate of enjoyment) differ according with the type of training sessions. As a consequence, we hypothesized that differences would exist between training types for the three above-mentioned parameters, lower pre-session well-being would be associated with higher RPE and session-RPE values and lower enjoyment, and that players’ and coach’s perceptions would emerge.

## Materials and methods

### Subjects

The institutional Ethical Committee of the University of Turin (protocol #0619881) approved the experimental design of the present study. In addition, written information on the potential risks and benefits associated with participation was provided to each participant who signed a written consent form. No minor participants have been recruited for this study.

Seventeen male master water polo players from *Torino 81* club (*Vintage team*; age: 50 ± 12 years, range = 35–65 years; stature: 180 ± 5 cm; body mass: 85 ± 12 kg) participated in the study performing 19 ± 7 (range = 12–36) individual training sessions. All participants competed in the Italian Master Water Polo Championship (i.e., > 30 years old players, not involved in other national or local agonistic federal Championship) [22] and had played in one of the top four national divisions at least 10 years prior to data collection. Over a 12-week period (outside of official competition periods), data were collected from 46 team training sessions: 13 SW (i.e., swimming repetitions according to time rest periods), 4 TTW (i.e., individual and team technical water polo patterns, with and without ball possession, also simulating specific phases of game), 26 TM (i.e., training session consisting of a match among opponent players of the same club, taking part in the experimental sample), 3 FM (i.e., friendly match played by players of the same club, taking part in the experimental sample, for a team, and opponent players of another club, not taking part in the experimental sample). Training sessions occurred on Mondays, Tuesdays, Thursdays, and Saturdays (generally during even hours, between 7 p.m. and 10 p.m.). Four goalkeepers and ten players were excluded due to their distinct training protocols and to have not perform a minimum of ten sessions within the experimental data collection, respectively.

### Design

The Hooper Index was employed to assess the player’s well-being status. Each player rated perceived sleep quality/disorders, stress level, fatigue, DOMS on a scale from 1 (very low/good) to 7 (very high/poor). The total score, obtained by summing the four ratings, provided an individualized measure of well-being measure before each training session [12].

The Italian translation of the CR-10 scale modified by Foster et al. [24] was used to assess the male master water polo players’ RPE. The CR-10 RPE scale, ranging from 0 (rest) to 10 (maximal exertion), is a category-ratio scale characterized by scores and verbal links that evaluates perceived effort in response to the question “How was your workout?”. According to literature [24], in this study, the RPE was administered to water polo players around 30 minutes after the end of each training session to assess the ITL of the entire training session, also allowing to calculate the session-RPE as product of RPE score and session duration expressed in minutes. Finally, water polo players rated their overall enjoyment of the training session on a 7-point Likert scale ranging from a minimum of 1 (i.e., “not at all enjoyed”) to a maximum of 7 (i.e., “extremely enjoyed”) [16].

### Methodology

Two online questionnaires were administered electronically via the Uniquest web platform on training session days. The first questionnaire, focused on the Hooper Index and was completed in the early morning (i.e., between 6 a.m. and 8.30 a.m. according to individual daily needs) to assess each player’s well-being status. The second, administered 30 minutes post-session (i.e., between 9.00 p.m. and 10.30 p.m. according to the session hour), collected data on RPE and enjoyment scores. Each session was classified according to the main training focus determined by the coach, and its duration was recorded. Three-week familiarization period was performed before the beginning of the study.

### Statistical analysis

Means and 95% confidence intervals (CIs) were calculated for all variables considered in the study. Data were reported for the total sample as well as stratified by training type. A series of linear mixed-effects models (LMMs) were applied to determine the impact of training type on RPE, s-RPE, and rate of enjoyment. To account for within-subject correlations due to repeated measures, players were included as random effects in all LMMs. Partial Eta Square (η_p_) were calculated to describe the practical meaningfulness of the differences in mean values. Post hoc pairwise comparisons with Tukey correction were conducted, when appropriate, to investigate differences among training types. Cohen’s d effect sizes (ES) with 95% were applied to describe the actual relevance of the differences in mean values. Absolute ES value was evaluated according to the following thresholds: < 0.2 = trivial, 0.2–0.6 = small, 0.7–1.2 = moderate, 1.3–2.0 = large, and > 2.0 = very large.

Additionally, in a subsequent step, variables related to well-being (i.e., sleep quality, stress, fatigue, DOMS, and Hooper index) were included in the models to further explore their influence. Standardized regression coefficients (β) were calculated using the pseudo-standardization method to allow comparison of the relative importance of predictors in the models. The level of signiﬁcance was set at 5% (P < .05). All data were analyzed using the statistical software R (version 4.3.0; R Core Team, Vienna, Austria) with the packages “lme4” for linear mixed-effects models and “emmeans” for estimated marginal means and pairwise comparisons.

## Results

### Players’ and coach’s ITL perceptions and rate of enjoyment

Descriptive values (estimate means ± standard errors, confidence intervals) of RPE, session-RPE, and rate of enjoyment, and differences between types of training according to players’ and the coach’s perceptions, were reported in Table 1.

**Table 1:** Estimate means ± standard errors, confidence intervals of RPE, session-RPE, and rate of enjoyment, and differences (p ≤ 0.05, p ≤ 0.01, p ≤ 0.001) between types of training according to players’ and the coach’s perceptions.

	Type of training session			
	Swimming (n cases = 90)	Technical and tactical (n cases = 20)	Training matches (n cases = 136)	Friendly matches (n cases = 13)
Players’ ITL perceptions				
RPE (range 0–10)	3.91 ± 0.2 ^**, ααα^ (3.4–4.4)	2.6 ± 0.4 ^ββ^ (1.8–3.4)	3 ± 0.2 ^ββ^ (2.5–3.4)	4.57 ± 0.5 (3.7–5.5)
Session-RPE (RPE*duration in min)	234 ± 16 ^βββ^ (202–267)	175 ± 26.4 ^βββ^ (122–227)	208 ± 14.6 ^βββ^ (178–238)	378 ± 31.6 (315–440)
Enjoyment (range 1–7)	3.2 ± 0.2 ^βββ^ (2.8–3.6)	4.1 ± 0.3 ^β^ (3.4–4.8)	4.3 ± 0.2 ^βββ^ (3.9–4.7)	5.2 ± 0.4 (4.4–6)
Coach’s ITL perceptions				
RPE (range 0–10)	4.9 ± 0.1^***, ααα^ (4.6–5.2)	3.5 ± 0.3 ^ββ^ (3–3.9)	3.8 ± 0.1 ^β^ (3.7–4)	4.8 ± 0.3 (4.2–5.5)
Session-RPE (RPE*duration in min)	295 ± 8 ^*, α, βββ^ (279–311)	241 ± 16.9 ^βββ^ (207–274)	264 ± 6.5 ^βββ^ (251–277)	397 ± 20.9 (356–438)
Enjoyment (range 1–7)	2.8 ± 0.1^***, ααα, βββ^ (2.6–2.9)	5.1 ± 0.1 ^α, βββ^ (4.8–5.4)	4.7 ± 0.1 ^βββ^ (4.6–4.8)	6 ± 0.2 (5.7–6.3)

* (p≤0.05), ** (p≤0.01), *** (p≤0.001) difference with respect to technical and tactical;

α p ≤ 0.05), ^αα^(p ≤ 0.01), ^ααα^(p ≤ 0.001) difference with respect to training matches;

β (p ≤ 0.05), ^ββ^(p ≤ 0.01), ^βββ^(p ≤ 0.001) difference with respect to friendly matches.

**Fig 1:**
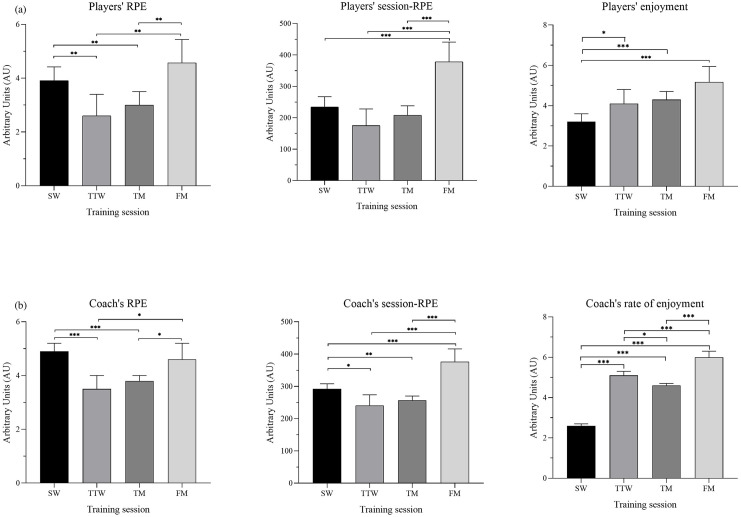
Estimated means and standard errors of RPE, session-RPE, and rate of enjoyment of water polo master players in relation to different types of training sessions (^*^p ≤ 0.05, ^**^p ≤ 0.01, ^***^p ≤ 0.001, difference between training session types).

The LMM applied to players’ RPE data (Fig 1a) revealed difference among training session types (F = 10.859; η_p_ = 0.12; p < 0.001). Post-hoc analysis indicated that RPE was higher in SW compared to TM (estimate mean difference (EMD)=0.94 arbitrary units (AU); p < 0.001, ES = 0.6) and TTW (EMD = 1.32AU, p = 0.004, ES = 0.9). Additionally, RPE during FM was higher than in TM (EMD = 1.59AU, p = 0.003, ES = 1) and TTW (EMD = 1.98AU, p = 0.002, ES = 1.3). Regarding session-RPE, the LMM analysis also showed differences (F = 11.69; η_p_ = 0.12; p < 0.001) with lower values in SW compared to FM (EMD = −143.7AU, p < 0.001, ES = 1.4), and higher values in FM compared to TM (EMD = 169.9AU, p < 0.001, ES = 1.6) and TTW (EMD = 203.1AU, p < 0.001, ES = 1.9). A similar pattern was observed for players’ rate of enjoyment (F = 15.203; η_p_ = 0.16; p < 0.001), with higher values for SW with respect to FM (EMD = 1.99AU, p < 0.001, ES = 1.4), TTW (EMD = 1.06AU, p = 0.049, ES = 0.7), and TM (EMD = 0.87AU, p < 0.001, ES = 0.8).

A different pattern emerged when analyzing coaches (Fig 1b). Regarding RPE, differences were observed (F = 21.25; η_p_ = 0.20; p < 0.001): RPE was higher in SW compared to TM (EMD = 1.07AU, p < 0.001, ES = 1) and TTW (EMD = 1.47AU, p < 0.001, ES = 1.3). Furthermore, FM showed higher RPE compared to TM (EMD = 0.99AU, p = 0.01, ES = 0.9) and TTW (EMD = 1.40AU, p = 0.003, ES = 1.2). Differences were observed for session-RPE (F = 15.71; η_p_ = 0.16; p < 0.0001), with lower values for SW than FM (EMD = −101.6 AU, p < 0.001, ES = 1.4), but higher with respect TTW (EMD = 54.6 AU, p = 0.02, ES = 0.7) and TM (EMD = 31.5 AU, p = 0.013, ES = 0.4); whereas FM reported higher session-RPE values than TTW (EMD = 156.2 AU, p < 0.001, ES = 2.1) and TM (EMD = 133.1 AU, p < 0.001, ES = 1.8). For the rate of enjoyment estimated by the coach (F = 247.9; η_p_ = 0.74; p < 0.0001), SW reported higher values than that FM (EMD = 3.22AU, p < 0.001, ES = 5.3), TTW (EMD = 0.90AU, p < 0.001, ES = 3.9), and TM (EMD = 1.32AU, p < 0.001, ES = 3.2), as well as TTW with respect to FM (EMD = 2.32AU, p < 0.001, ES = 1.5) and TM (EMD = 0.42 AU, p = 0.02, ES = 0.7), and TM with respect to FM (EMD = 1.91 AU, p < 0.001, ES = 2.2).

### Well-being parameters, ITL perceptions, and rate of enjoyment

The LMMs applied to verify the interactions between well-being (Table 2) factors and players’ RPE showed effects for stress (β = 0.16), fatigue (β = 0.28), DOMS (β = 0.19), and Hooper index total score (β = 0.28). No effect was found for sleep quality/disorders. More specifically, players’ RPE resulted negatively associated with stress for TM (β = −0.30) and TTW (β = −0.23); negatively associated with fatigue for TM (β = −0.29) and technical tactical workouts (β = −0.24); negatively associated with DOMS for TM (β = −0.29) and technical tactical workouts (β = −0.24) and negatively associated with Hooper index total score for TM (β = −0.29) and technical tactical workouts (β = −0.24).

**Table 2:** Outcomes of Linear Mixed-Effects Models (β) applied for players’ well-being parameters, in different types of training session (compared with the swimming session), and in relation to the RPE, Session-RPE, and rate of enjoyment.

Well-being Parameters	RPE				Session-RPE				Rate of enjoyment			
	β	SE	b	CI	β	SE	b	CI	β	SE	b	CI
**Sleep disorders**												
Friendly matches	−0.65	0.46	0.09	(−0.03, 0.21)	−143.1***	31.70	0.28	(0.16, 0.40)	−1.99***	0.42	0.29	(0.17, 0.41)
Training matches	0.94***	0.21	−0.29	(−0.42, −0.16)	26.60	14.60	−0.12	(−0.25, 0.01)	−1.12***	0.19	0.37	(0.25, 0.50)
Technical & tactical workouts	1.31**	0.39	−0.22	(−0.34, −0.09)	59.00	26.70	−0.14	(−0.27, −0.02)	−0.93	0.35	0.17	(0.04, 0.29)
**Stress**												
Friendly matches	−0.71	0.46	0.10	(−0.03, 0.22)	−146.5***	31.70	0.29	(0.16, 0.41)	−1.96***	0.42	0.29	(0.17, 0.41)
Training matches	0.96***	0.21	−0.30	(−0.43, −0.17)	27.40	14.60	−0.12	(−0.25, 0.01)	−1.13***	0.19	0.38	(0.25, 0.50)
Technical & tactical workouts	1.40**	0.39	−0.23	(−0.36, −0.11)	63.80	26.80	−0.15	(−0.28, −0.03)	−0.97*	0.36	0.17	(0.5, 0.30)
**Fatigue**												
Friendly matches	−0.80	0.45	0.11	(−0.01, 0.23)	−152.2***	31.30	0.30	(0.18, 0.42)	−2.03***	0.42	0.30	(0.18, 0.41)
Training matches	0.92***	0.20	−0.29	(−0.41, −0.16)	25.50	14.40	−0.11	(−0.24, 0.01)	−1.12***	0.19	0.37	(0.25, 0.50)
Technical & tactical workouts	1.42**	0.38	−0.24	(−0.36, −0.11)	65.10	26.30	−0.16	(−0.28, −0.03)	−0.91	0.35	0.16	(0.04, 0.28)
**DOMS**												
Friendly matches	−0.77	0.46	0.10	(−0.02, 0.23)	−149.7***	31.80	0.29	(0.17, 0.41)	−1.96***	0.42	0.29	(0.17, 0.41)
Training matches	0.92***	0.21	−0.29	(−0.41, −0.16)	25.50	14.50	−0.11	(−0.24, 0.01)	−1.11***	0.19	0.37	(0.25, 0.50)
Technical & tactical workouts	1.42**	0.38	−0.24	(−0.36, −0.11)	64.70	26.80	−0.15	(−0.28, −0.03)	−0.96*	0.35	0.17	(0.05, 0.29)
**Hooper Index**												
Friendly matches	−0.78	0.45	0.11	(−0.1, 0.23)	−150.5***	31.40	0.29	(0.17, 0.41)	−1.98***	0.42	0.29	(0.17, 0.41)
Training matches	0.95***	0.21	−0.29	(−0.42, −0.17)	26.80	14.40	−0.12	(−0.25, 0.01)	−1.12***	0.19	0.37	(0.25, 0.50)
Technical & tactical workouts	1.44**	0.38	−0.24	(−0.36, −0.12)	66.20	26.50	−0.16	(−0.28, −0.03)	−0.94*	0.36	0.17	(0.04, 0.29)

* (p≤0.05), ** (p≤0.01), *** (p≤0.001) difference with respect to swimming.

Regarding the influence of well-being factors on players’ session-RPE, a general positive influence was found between s-RPE and fatigue (β = 0.23). Specifically, after FM, fatigue had a positive influence (β = 0.30), while after technical tactical workouts fatigue showed a negative association (β = −0.16). A positive influence was found between s-RPE and Hooper index total score (β = 0.23). In details, after FM, HI influence positively (β = 0.29), and after technical tactical workouts HI influence negatively (β = −0.16). No associations were found between players’ rate of enjoyment and any of the individual well-being components.

### Comparison between players’ and coach’s perceptions

LMMs applied on RPE and rate of enjoyment for comparing corresponding players’ perceptions and coach’s estimations in relation to types of training sessions, reported no significant correlation for SW, TTW, TM, and FM. Only for sRPE, comparing players’ and coach’s perceptions, an effect in TM was founded with a positive correlation (β = 0.21).

## Discussion

Although studies on master athletes training and competing at high levels is increasing [25] research on a sample of water polo master players is limited [26]. To our knowledge, this is the first study to quantify ITL (i.e., RPE and session-RPE) and rate of enjoyment in a sample of athletes of this type, considering different training session (generally with moderate or large ESs), their relationships with pre-session well-being (Hooper Index), and the relationships between players’ and coach’s perceptions. The main findings indicate that FM elicited the highest ITL and rate of enjoyment. Conversely, TTW showed the lowest ITL, and SW was associated with the lowest rate of enjoyment. Furthermore, independently to training sessions, stress, fatigue, and DOMS generally influenced players’ post-session RPE, whereas session-RPE was affected only by fatigue and the overall well-being score (Hooper Index). Finally, no strong correlation was found between players’ perceptions and coach’s estimations in terms of both ITL or enjoyment indexes.

Data showed how SW and FM represent the highest players’ exertion among sessions. These results are partially consistent with previous findings reported for youth water polo players [3] where only SW were associated with the highest RPE values. The difference may arise in session classification, as the present study distinguishes FM as a separate category. RPE values reported after FM (i.e., not official matches, played against different clubs) are higher than TTW and TM (against players of the same club), supporting earlier evidence that highlighted how heart rate is higher during FM than TM [27].

Although RPE and session-RPE are related, the shorter duration of the SW explains why only FM have higher values than those of other training types in line with findings from other team sports [9]. Moreover, heightened focus on opponents may cause athletes to underestimate their actual exertion relative to physiological demands [28]. Consequently, the ITL parameters recorded after the FM might represent an even higher training load than what was reported by the athletes’ perception, with a probable less recovery capability [19]. Even though players’ rate of enjoyment may show different patterns compared to ITL values, FM showed higher enjoyment levels than other types of training sessions. These results align with previous findings in football [29] where enjoyment increases during technical and tactical drills (involving ball possession) compared to cardiovascular training sessions (without ball possession), and can contribute to the scenario already defined by Perazzetti et al. [19] for youth water polo players, in which enjoyment is higher after playing official matches than FM ones. Thus, it can be inferred that open-skill water polo workouts and playing tasks provide valuable opportunities to engage players at high internal training loads (ITLs) while also promoting higher enjoyment. This, however, makes it more complex to determine whether conditioning or open-skill (e.g., technical or tactical) sessions are truly the most effective in enhancing players’ performance [30].

Finally, the coach’s ITL ratings followed similar players’ trends, also identifying SW and FM as higher values compared to TTW and TM, thus confirming, only for SW, the same trend of youth water polo players [3]. However, almost all analyzed players’ RPE, session-RPE, and enjoyment and corresponding coach’s estimations are not correlated, confirming the main findings reported in other sport environments for the same comparison [10]. In particular, players rates SW slightly lower in ITL than coaches, placing this type of training in a moderate range from the players’ perspective. However, despite this slight divergence, both perspectives aligned on enjoyment ratings, identifying SW as the least enjoyable and FM as the most enjoyable training units. This observation further suggests varying degrees of post-training recovery, consistent with previous findings on water polo post-matches [7].

For the second aim of this study, well-being factors such as stress, fatigue, and DOMS, as well as the overall HI score, directly influence players’ RPE levels, thus highlighting how reduce scores in these factors (i.e., high wellbeing level) can minimize the RPE after training session. Differently, only fatigue and overall HI scores are positively correlated to session-RPE. These trends seem to align with earlier research on soccer players, where well-being status substantially influenced RPE [31]. Similarly, other studies found that pre-session well-being factors, particularly perceived fatigue, influence session-RPE in female volleyball players [15] whereas DOMS, perceived stress, sleep quality, and fatigue were associated with ITL in soccer [13] and American football [14]. In contrast, our findings suggest that sleep quality/disorders had no impact on ITL perceptions of male master water polo players due to the long interval (i.e., 11–13 hours) between well-being assessment and training.

When training sessions were analyzed separately, TTW both RPE and session-RPE showed a negative association with fatigue and overall Hooper Index scores, suggesting that poorer well-being may negative impact on ITL. This finding suggests that reduced well-being status may limit players’ ability to train at their highest capacity. In contrast, a different trend was observed in FM: higher fatigue and Hooper Index scores resulted associated to higher session-RPE, but not to RPE ones, probably due to its high mean session duration (FM: 83 ± 22 min; TM: 69 ± 9 min; SW: 60 ± 0; technical-tactical: 66 ± 8 min). Again, a significant relationship between well-being and ITL emerged for SW, indicating limited influence of pre-session status on perceived exertion. For TM, minimizing these two well-being factors seemed to allow higher RPE values. However, no effect was observed on session-RPE, indicating that RPE may more accurately capture peak exertion and correlate with pre-session status, whereas session-RPE is more sensitive to session duration [32].

Similarly, for stress, DOMS, and sleep disorders, significant correlations emerged only for RPE, and not for session-RPE, suggesting how picks of intensity, that might be represented better by RPE than session-RPE, tends to be highly related to pre-session status. Finally, in regarding with the second aim, no correlation was found between well-being factors and the rate of enjoyment, suggesting that the rate of enjoyment is strongly influenced by the type of training session but not by well-being pre-session condition. Even though our study considered well-being before session and rate of enjoyment after that, this result seems to be in contrast with a previous study on Italian first division water polo players [7], in which higher enjoyment values, perceived after playing official matches, were associated to a better well-being status, recorded at the beginning of the following training week.

The third aim of the study was to analyze the correlation between players’ perception and coaches’ estimations (i.e., RPE, session-RPE, and rate of enjoyment) across different types of training sessions. The analysis revealed that coach slightly overestimated their ratings compared to players’ perceptions, likely because the coach does not account for breaks that each player has inside the same training session (i.e., when the ball is not in player’s playing zone during TTW). In general, our findings are quite in line with previous studies [10,33], where coaches of several team sports tend to differently quantify ITL with respect to players.

The main limitation of this study was evaluating the ITL of water polo players without a more objective measure such as heart rate or external load parameters (e.g., swimming distance or workout repetitions). Moreover, RPE and rate of enjoyment could be affected by potential self-report biases which evidently limit any generalization, especially for coach category which has been represented by only one subject, reason of the high ESs emerged for this part of analyses. In addition, these master-level water polo players, as non-professional athletes, are characterized by a reduced number of weekly training sessions (not more than 3 or 4), by a high heterogeneity among players in terms of attending training sessions and competitive level and sport background. Therefore, other studies on training monitoring for this category of athletes, even regarding other team sports, could substantially contribute to better understand this growing sporting context [26].

From a practical perspective, these findings offer master coaches a valuable reference for evaluating the readiness and physical and mental status of their players. In particular, they underscore the influence of players’ well-being factors on perceived ITL across different training types and enjoyment levels. As a consequence, a regular use of Hooper Index in the morning of the training day represents a valuable monitoring that could lead to an eventual adjustment in training plans, even for the following session in the same day, thus tending to provide a progressive balance between players’ current capability and training challenge. Practically, coaches will be aware that FM and TTW represent the highest and lowest ITL respectively, and that SW represents the most boring session (especially from the coaches’ perspective). Furthermore, the observed tendency of coaches to overestimate ITL compared to players’ self-reports emphasizes the importance of integrating athlete feedback for more accurate training monitoring. Therefore, coaches should be aware that subjective well-being may not reliably predict post-session enjoyment or exertion perception. In addition, discrepancies between coach and player RPE suggest the need for ongoing communication.

## Conclusions

Results highlighted that different training sessions are discriminable in terms of RPE, session-RPE, and rate of enjoyment levels, with SW linked to lower enjoyment and higher exertion, while TM elicited both higher enjoyment and perceived load. Additionally, our results identified how pre-session well-being factors (especially fatigue and Hooper Index overall) affected post-session ITL levels. Finally, the lack of correlation between ITL players’ perceptions and coaches’ estimations also suggests how players’ perceptions have to be considered by coaches, even though they could be not perfectly reliable.

## Supporting information

S1 Dataset Minimal dataset used for statistical analyses. (XLSX)
